# The Maryland State Veterinary Medical Society

**Published:** 1894-10

**Authors:** A. W. Clement

**Affiliations:** 916 Cathedral St., Baltimore, Md.


### MARYLAND STATE VETERINARY MEDICAL ASSOCIATION.

The regular quarterly meeting of the Association was held at the Hotel Renert, on Tuesday, July 24th, at 2 P. M. In the absence of the President and Vice-President, the Secretary called the meeting to order. Dr. William H. Dougherty was elected chairman pro tempore. Drs. Dougherty, Lloyd and Clement responded to the roll call. Dr. Robert Ward was present as a visitor.

The Secretary read a letter from Dr. Stuart E. Paulet, tendering his resignation from the Association, as he was not willing to abide by the code of ethics. It was voted to accept Dr. Paulet's resignation. The Secretary also read a letter from the Secretary of the Virginia State Association, extending an invitation to this Association to send delegates to the meeting at Norfolk in August. It was voted to thank the Secretary of the Virginia Association for his kindness, but to say that it would be impossible for us to count on sending delegates to represent our society at Norfolk.

Dr. Clement read a paper on the History and Purposes of the Association, which was well received by the members present.

He said :—

MR. PRESIDENT AND GENTLEMEN :

This, the first assembling of our members under the system of quarterly meetings, while it may not and probably will not be as fully attended as might be wished, still in my opinion marks an era of progress, and affords a most fitting opportunity for pledges of support and enthusiasm by every veterinarian eligible for membership. If we will but each of us pledge ourselves to make the meetings profitable by imparting our knowledge to the common good, even though but three of us may be gathered together, the benefits to each will be of inestimable value. As one who has been honored with the position of Secretary, I feel that in accepting the position I have assumed a great responsibility, and one which can be of benefit to the Society only through the most interested assistance of all of the members. The number of veterinarians is constantly increasing in our State, and there is no excuse for lack of material. With our recent legislation providing against further entrance into our ranks of persons questionably educated, I think we can wisely so modify our by-laws as to admit all graduated veterinarians, who are registered as qualified to practice.

I wish to say here that out of three practitioners graduated whom I have asked to become members of this association, two of them have declared themselves ineligible from having graduated at schools which, by our by-laws, we have debarred from representation here. Whether we should not modify our by-laws to meet these men, is a question which should be decided here to-day. I think that I have some especial right to speak upon this subject, because, if I remember rightly, I was the mover or seconder of a resolution to make ineligible to membership graduates of certain schools, because said schools did not, in our opinion, aim to make better the profession by affording a means of obtaining such an education as would benefit the public to the extent which a college diploma should guarantee. I am certain that the colleges are impressed with the importance of extending their course of study, and I believe that they are being brought to realize their position the more fully from the demand made by the U. S. Society as a standard for entrance. The State Law which we had passed at Annapolis, while it is not as binding on the whole State as we all wished, is nevertheless based upon the standard required by the U. S. Society. It is the intention of the Examining Board to enforce the law, and it seems to me that the present is a fitting time for a general awakening of enthusiasm in our Association. I would, therefore, recommend that all registered graduated veterinarians be eligible for membership to our Association, and that our associate membership be increased by soliciting the co-operation of some of our fellow practitioners who, through no fault of their own, have not had the benefit of a college education.

I have met with many of the latter class, as doubtless have all of you, who would be encouraged by the recognition of this Society, and from whom all of us could learn many facts, as well as in turn impart to them useful information. For my part, I would much prefer a society many of whose members were non graduates but who were in earnest, than a society whose membership was composed entirely of graduated men who are either too ignorant or too indolent to enjoy the advantages of active participation in the transactions of such a society as this.

If after repeated solicitation, our members do not attend, and attending do not participate in the reading of papers and discussion of subjects brought before them, then it is time to drop their names from the list of membership, and fill their places with men who are professedly of a lower standard but who perhaps actually possess more intellectual merit, and who are not so pressed with professional cares and responsibilities that they have no time to learn from others or to impart a grain of information to those who are not so fortunate in the opportunity of accumulating practical experience from a very extended clientele.

Our Association, Mr. President, is one of the oldest, and, though its career has been rather varied, can well be proud of its existence. According to the records in my possession as Secretary, I find that the first call for a meeting was made in 1885, and that the first meeting was held on Monday evening, Sept. 28th, of that year. Drs. Dougherty, W. H. & A. H. Rose, Miller of Camden, Moulton, Steck, Sprank-lin and Martinet were present. All present joined the new Society. Officers were elected, and at the third meeting the Constitution and By-Laws were presented and adopted, and on Oct. 20th Articles of Incorporation, as follows :

### CERTIFICATE OF INCORPORATION OF MARYLAND STATE VETERINARY MEDICAL SOCIETY.

*Know all Men by these Presents, that we,*

| | | |
|---|---|---|
| William Dougherty, | Wm. H. Martenet, | Geo. M. Steck, |
| T. W. Spranklin, | A. H. Rose, | W. H. Rose, |

And C. L. Moulton,

Being citizens of the United States, and a majority of whom are citizens of the State of Maryland, do hereby certify that we do, under and by virtue of the General Laws of this State, authorizing the formation of Corporations, hereby form a Corporation under the name of *The Maryland State Veterinary Medical Society* (of Baltimore City).

We do further certify that the said Corporation so formed is a *Corporation for the Advancement of Veterinary Science;* that the term of existence of the said Corporation is limited to forty (40) years, and that the said Corporation is formed upon the articles, conditions and provisions herein expressed, and subject in all particulars to the limitations relating to corporations which are contained in the General Laws of the State.

We do further certify that the operations of the said Corporation are to be carried on in the State of Maryland, and that the principal office of the said Corporation will be located in Baltimore City.

We do further certify that the said Corporation will be managed by a Board of Censors, and that Wm. Dougherty, T. W. Spranklin, Geo. M. Steck, A H. Rose and W. H. Rose are the Board of Censors who will manage the affairs of the said Corporation for the first year.

In witness whereof, we have herewith set our hands and seals this 20th day of October, in the year eighteen hundred and eighty-five.

Test:

STEPHEN S. CLARK.

WM. DOUGHERTY, D. V. S. [SEAL]
WM. H. MARTENET, D. V. S. [SEAL]
GEO. M. STECK, D. V. S. [SEAL]
CHARLES L. MOULTON, D.V. S. [SEAL]
THOMAS W. SPRANKLIN, D.V. S. [SEAL]
ALVORD H. ROSE, D.V. S. [SEAL]

STATE OF MARYLAND,
BALTIMORE CITY, TO WIT:

Before the subscriber, a Justice of the Peace of the State of Maryland, in and for the city of Baltimore, personally appeared, on the 20th day of October, in the year of eighteen hundred and eighty-five, William Dougherty, Wm. H. Martenet, Geo. M. Steck, Charles L. Moulton, Thomas W. Spranklin and Alvord H. Rose, and did severally acknowledge the foregoing certificate to be their act and deed.

STEPHEN S. CLARK, J. P.

I, George William Brown, one of the Judges of the Supreme Bench of Baltimore City, do hereby certify that the foregoing certificate has been submitted to me for examination, and I do further certify that the said certificate is in conformity with the provisions of the law authorizing the formation of said Corporation.

GEO. WM. BROWN.

It was within eight days of nine months after its organization before a paper was offered for the consideration of this society, although informal discussions appear to have been indulged in frequently. Then, as now, do we see the names of members who promised to read papers at the next meeting, but in some cases the next meeting has to them apparently never come. Dr. W. H. Wray, had the honor of reading the first paper, one on contagious pleuro-pneumonia, which was well received and gave rise to a general discussion of the subject. There have been seventy-five meetings of our Association held, but at only sixty-six was there a roll call, and some of the meetings were not even recorded. The average attendance at the sixty-six meetings at which the roll was called was six. The average membership has been about ten, confined almost exclusively to practitioners in Baltimore City, and including those associated with the Bureau of Animal Industry. At thirty-four of the sixty-six meetings reported, papers were read or demonstrations given. Many of our meetings have been most interesting and instructive. Most of us present remember well the interesting meetings held by lamp light in the third story of the building on St. Paul Street, at the office of the inspectors of the Bureau of Animal Industry, and later, when we met in the hall of the Medical Chirurgical faculty. We all seemed to be enthusiastic then—why should we not be now, when the material is greater, and the facilities for obtaining information growing year by year? Why should we not, with the ever-increasing sources of information, come prepared, each and every one of us, at every meeting to offer some information from our store of experience for the benefit of our fellow members? It matters not so much how limited our membership, if we but methodically set to work to obtain the greatest possible information from each other by honestly and modestly laying our observations before the meeting, thereby inviting discussion. I sometimes think it a pity that we did not at first assume the more modest, and to my mind more appropriate title of Society, unless we should anticipate an aggressive tone in the way of future legislation. Such has not been our method so far, and I am inclined to hope never will be. Rather have we aimed to assist as a body the profession at large in any movement toward the public good, and I think that in a great measure we have succeeded.

To be sure, we have in many cases not accomplished all that we wished, but I am certain that we have made our influence felt, and if we but keep on, we will reap the fruits of our reward. We have held meetings, and gone to considerable trouble and some expense to inform the public of the danger lurking in the meat and milk supply of our city and State. We have shown the necessity of veterinary experience of the City and State Boards of Health, and we assisted with our money and our time in initiating the measure which resulted in legislation for the protection of our profession. I am fully of the opinion that our agitation of the question of a supervision of the milk and meat supply has had a great deal to do with the appointing of inspectors, worthless though they may be in our eyes, and, perhaps, probably worse than useless so far as the consumers are concerned, but it is a beginning, and if the public in the future do not care for anything better, it is not our fault, and we can probably stand it. I am convinced that, however, the time is coming when better things will be demanded, and when the public really demands a thing in this country, they generally get it. Americans are not slow of observation, and it is as certain that trouble will follow the appointment of inexperienced men to go about deciding whether milk is deadly or not simply by putting a register into the fluid, which they are taught mechanically to read, or to decide on the merit of meat by its smell, as it is that a child will get cut if it plays with sharp instruments. The practice of appointing inexperienced men to such a position as inspector of meat and milk is a fraud upon the public, a gross injustice to the producer, and an insult to our profession. Yet I would not be one to publicly proclaim this fact, for I believe that our influence will be of greater weight by the better education of the public and our public officials to the importance of the question, in a quieter and less aggressive manner. The time is sure to come, in the near future, when some of us will be called upon to give our opinion fearlessly and honestly. Then it will become our duty and our privilege to uphold that wich is good and condemn that which is bad in the present system. Of how much more weight, Mr. President and gentlemen, will be our opinion if we have taken the precaution to fortify ourselves with the knowledge best gained by mutual intercourse, the expression of opinions gained by discussion based upon observation and extended reading.

How can we do this, except by a systematic gathering such as this? Yes, gentlemen, there is reason for the existence of such a society as this, and every veterinarian who is eligible to membership makes a very grave mistake, in the opinion of the best element of the profession throughout the world, in not taking advantage of it. We should respect ourselves and respect each other; then, and not until then, will the people respect us and heed our warnings. We must go on, and we must awaken enthusiasm among our members. It is not to be expected that every one of us can attend each meeting, such a thing would be impossible, but the same ones should not be absent continuously, and the same ones always present.

If we have grievances, let us make them known, and if they are not corrected, let us assert ourselves at the annual election of officers. We have shown more than once what we can do. We gave the United States Society as good a reception as they ever had anywhere, and we held one of the best meetings here, which up to that time had ever been held. We often have as large a representation at the meetings of the United States Society as we have attendance at some of our State meetings.

We have the reputation in that Society of being enthusiastic and working in harmony. At the same time, they must wonder why they never see any mention of our existence in the journals. But such lack of enthusiasm, so well known to us all, of late, is not to exist longer, I am sure, for there is no reason in it, and it only needs a little exertion on the part of each one of us to make our Association a grand success.

The meeting then adjourned.

A. W. CLEMENT,

916 Cathedral St., Baltimore, Md.

July 24th, 1894.

